# The potential impact of exercise upon symptom burden in adolescents and young adults undergoing cancer treatment

**DOI:** 10.1007/s00520-024-08497-0

**Published:** 2024-04-17

**Authors:** Claire Munsie, Jay Ebert, David Joske, Jo Collins, Timothy Ackland

**Affiliations:** 1 School of Human Sciences (Exercise and Sport Science), University of Western Australia, Perth, Western Australia Australia; 2 WA Youth Cancer Service, Locked Bag 2012, Nedlands, Perth, Western Australia 6009 Australia; 3 Sir Charles Gairdner Hospital, Perth, Western Australia Australia; 4 School of Medicine, University of Western Australia, Perth, Western Australia Australia

**Keywords:** Exercise intervention, Rehabilitation, Health and well-being, Cancer care continuum, Treatment-related toxicities

## Abstract

**Purpose:**

Adolescents and young adults (AYAs) experience vast symptom burden resulting from cancer treatment-related toxicities (TRTs). Evidence supports integrated exercise to mitigate several TRTs in other cohorts; however, evidence in AYAs is lacking. Conventional reporting of TRTs adopts a maximum grade approach failing to recognise the trajectory over time, of persistent, or lower grade toxicities. Alternatively, longitudinal analysis of toxicities over time (ToxT) may provide clinically meaningful summaries of this data. We evaluated the longitudinal impact of an exercise intervention on TRTs in AYAs undergoing cancer treatment.

**Methods:**

A prospective, randomised trial allocated participants to a 10-week exercise intervention (EG) or control group (CG) undergoing usual care. Detailed information on TRTs was collected throughout the intervention. All TRTs were graded per the Common Terminology Criteria for Adverse Events (CTCAE v5.0).

**Results:**

Forty-three (43) participants (63% male, mean age 21.1 years) were enrolled. When categorised to reflect the maximal worst grade experienced (Grade 0, Grade 1–2 and ≥ Grade 3), the CG reported an increased incidence of severe fatigue (≥ Grade 3) compared with the EG (*p* = 0.05). No other differences between groups were evident (*p* > 0.05). ToxT analysis of the four most common toxicities (fatigue, pain, nausea and mood disturbances) demonstrated no difference in the mean grade of each over time (*p* > 0.05).

**Conclusion:**

A 10-week exercise intervention reduces the severity of fatigue in AYAs undergoing treatment. While the ToxT approach provided insight into the toxicity profile, adequately powered studies are needed to better understand these differences within a homogenous sample.

**Trial registration:**

(ACTRN12620000663954) 10^th^ June 2020.

**Supplementary Information:**

The online version contains supplementary material available at 10.1007/s00520-024-08497-0.

Adolescents and young adults (AYAs) with cancer face unique challenges, both physically and psychosocially, from their diagnosis throughout and after treatment. Over the last three decades, 5-year survival rates in this cohort have improved through adaptations in treatment protocols, development of new therapies and improvements in supportive care [1, 2]. The increasing use of intense treatment protocols, traditionally used in paediatric cohorts, has led to AYAs experiencing an increased frequency and intensity of treatment-related toxicities (TRT) resulting in an amplified symptom burden comparative to younger and older cohorts [2–4]. Effective supportive care strategies are needed, primarily aimed to assist with the management of such toxicities in reducing the burden of cancer in this cohort.

The highest symptom burden in AYAs has been attributed to fatigue, nausea and pain, with these physical symptoms being a significant predictor of health-related quality of life (HRQOL) [5, 6]. While not often dose-limiting, these side effects, coupled with peripheral neuropathy, sarcopenia, physical deconditioning, prolonged neutropenia and unplanned hospitalisations, can lead to alterations in treatment protocols [6–8]. Ultimately, any significant TRT that results in protocol deviations, may be a prognostic risk factor for recurrence and has the potential to negatively impact upon survival outcomes [6, 9].

Conventional methods for reporting TRTs in clinical studies narrowly focus on the incidence of severe or life-threatening events experienced by an individual at a single time point throughout their treatment (≥ Grade 3) [10]. This one-dimensional approach fails to recognise the time course of TRTs or the burden of lower grade (< Grade 3), chronic or persistent toxicities, which often worsen a patient’s ability to tolerate their treatment long-term and are subsequently associated with poorer QOL. In recent years, the toxicity over time approach (ToxT) has been successfully adopted across multiple cohorts [10]. This uses multiple longitudinal analysis methods and graphical representations of TRTs collected over time, providing a comprehensive, statistical illustration of symptom burden.

In recent decades, exercise as a toxicity management tool in cancer cohorts has begun to be explored [11]. There is growing evidence that exercise is safe, feasible and effective in the management of several TRTs [12–14]. Robust data support the benefit of exercise in the management of cancer-related fatigue, anxiety and depression, lymphoedema and HRQOL in a range of adult cancer populations [13]. However, no high-quality randomised controlled trials (RCTs) have reported on TRTs relative to exercise interventions in AYAs [15]. Additionally, to our knowledge, there are no clinical trials evaluating the impact of exercise relative to TRTs collected using the CTCAE. Therefore, we sought to investigate the impact of a supervised exercise intervention on patient-reported symptomology TRTs specifically in AYAs.

## Methods

### Participants

**Fig. 1:**
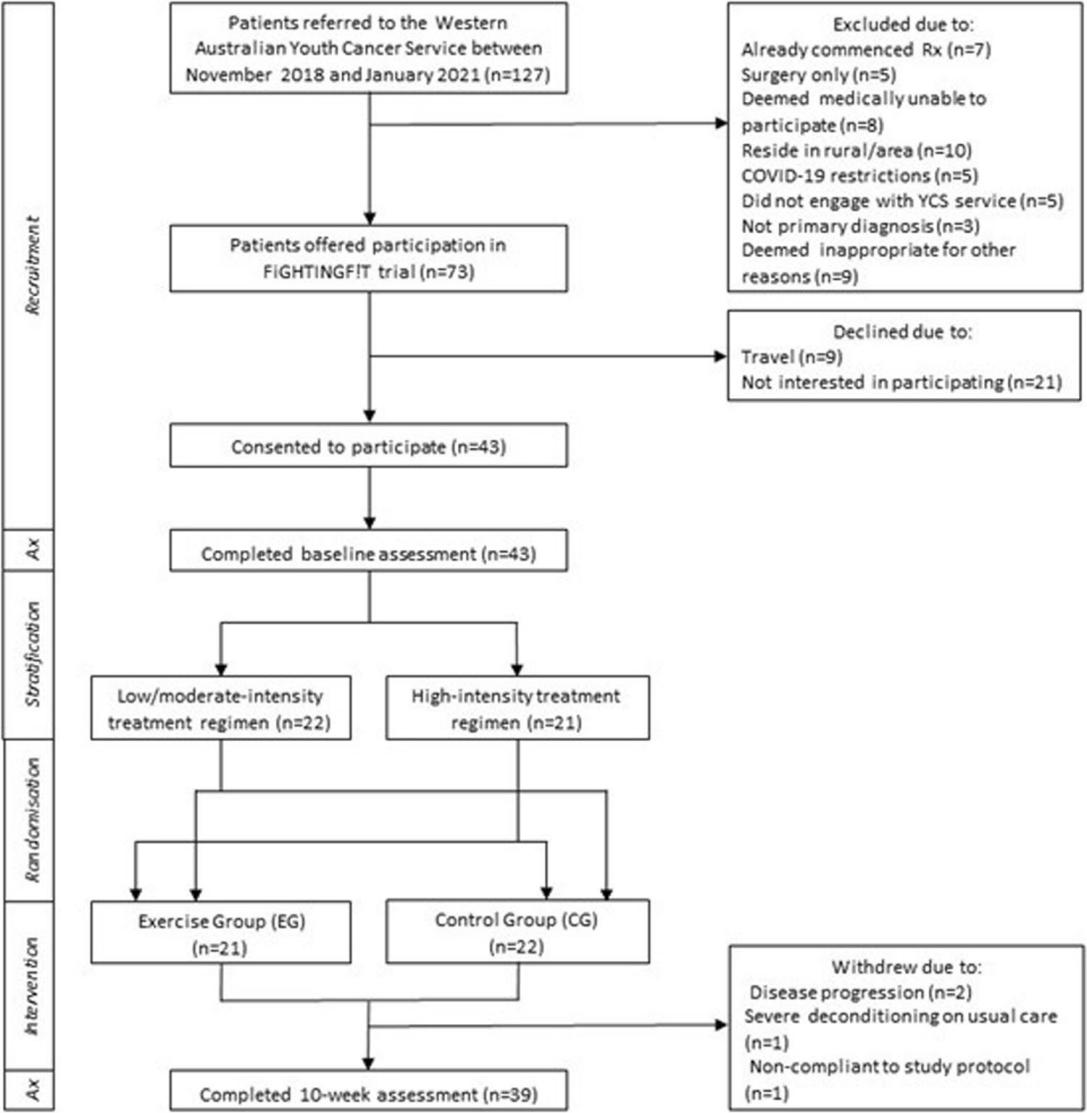
Flowchart of participant recruitment

A total of 127 AYAs aged 15–25 years diagnosed with cancer were referred to the Western Australian Youth Cancer Service (WAYCS) from November 2018 to January 2021 and screened for participation in the study (Fig. 1). Participants were eligible if their diagnosis was a primary malignancy, they were medically stable as per their treating clinician and were assessed (within 2 weeks) prior to commencing systemic therapy (e.g. chemotherapy or combined chemotherapy and radiation). Participants were excluded if they were to undergo surgery only, had insufficient English competency or a cognitive impairment that would prevent them from participating in the programme, were medically unable to participate (as determined by their treating clinician) and were pregnant or lactating or had a life expectancy < 6 months. The study was conducted in accordance with the Declaration of Helsinki and was approved by the relevant University and Hospital Human Research Ethics Committees (Protocol Version 2. 03012019). All participants and their treating clinician provided written informed consent to participate in this study. This trial was registered with the Australian New Zealand Clinical Trials Registry (ACTRN12620000663954).

### Experimental design

A prospective, single-centre RCT design was implemented. Participants were stratified to reduce bias, according to the intensity of their treatment regimen; low/moderate or high intensity treatment. Stratification was overseen by a treating clinician and based on the likelihood of myelosuppression anticipated from the treatment regimen (Supplementary material 1) [16]. Randomisation was undertaken via a random assignment computer generator (sealed envelope) into one of the two treatment arms: exercise group (EG) or control group (CG). All participants completed a series of assessments at baseline and at 10 weeks following the intervention.

### Exercise (EG) and control (CG) group interventions

The CG received general physical activity advice from the WAYCS Accredited Exercise Physiologist (AEP), the broader WAYCS team and their treating clinical team as part of standard care; however, no exercise intervention was offered. The EG involved twice weekly exercise sessions for 10 weeks at a purpose-built gymnasium in a central hospital location. Sessions were individualised to each participant’s needs based on the results of their functional ability captured through baseline assessments. Sessions were approximately 60 min in duration, in a one-to-one setting and supervised by an AEP. Each session was a combined aerobic, resistance and flexibility exercise programme of moderate intensity as per the American College of Sports Medicine (ACSM) guidelines for cancer cohorts [17]. Sessions began with a standardised 5-min aerobic warm-up, followed by a series of strength-based exercises and finished with a low intensity 5-min aerobic cooldown. A series of flexibility exercises were included in the cooldown targeting major muscle groups. Aerobic exercise was completed following the warm-up and prescribed at 60–85% of maximal heart rate (220 age) [18] and for up to 30 min duration (inclusive of warm-up and cooldown), including stationary cycling, walking, skipping and/or jogging. The strength-based component included eight resistance exercises that targeted each of the major muscle groups within the body. Body weight, dumbbell and machine-based exercises were utilised and prescribed at 60–80% of one-repetition maximum (1RM) as determined initially by participants’ baseline functional assessment results. The Borg scale for perceived exertion was utilised throughout the session with the aim to maintain intensity at 12–15 (out of 20) units [19].

Prior to commencing each exercise session, the participant’s most recent full blood count results were reviewed to determine their safety for exercise [20]. Based on these results, as well as any other treatment-related side effects, programme modifications were adopted if necessary. Participants were instructed to attend their usual medical follow-ups, as well as continue their usual physical activity and dietary intake throughout the intervention period. Additionally, all participants were required to complete an activity journal to capture any incidental exercise completed throughout the course of the trial, and the contents of the journal were reviewed on a weekly basis by the study team.

### Outcome measures

#### Patient-reported TRTs

Toxicity data were collected weekly over the 10-week intervention period. Participants were contacted by a member of the research team blinded to group allocation to determine if they had experienced any TRTs including, but not limited to, nausea, vomiting, constipation, pain, peripheral neuropathy, mucositis and fatigue within the previous week. All TRTs were also recorded in the participants’ medical records and relayed to treating clinicians if deemed necessary.

Toxicities were graded as per the Common Terminology Criteria for Adverse Events (CTCAE Version 5.0) [21]. The CTCAE is widely accepted as the standard classification and severity grading scale for adverse events related to cancer treatments. The severity of an adverse event is graded from 1 (mild) to 4 (hospitalisation) or 5 (death), with grades 3 and 4 the most likely to result in modifications to planned treatment protocols. TRTs were captured across all grades.

### Statistical analysis

Toxicity data were analysed using both conventional (maximum worst grade) and ToxT approaches. Participants were classified into three groups, Grade 0, Grade 1–2 and ≥ Grade 3, based on the maximum grade experienced for each variable at any point throughout the 10-week period. This classification was selected both to align with standard reporting (maximal worst grade) and lower-grade toxicities’ reporting. For each variable, the worst grade recorded over the 10-week period was used for this analysis. Participants were coded as 0 (Grade 0), 1 (Grade 1–2) and 2 (Grade 3–4) representing the highest toxicity experienced. Data were then reported as the frequency and proportion of the sample in each group for each variable.

Preliminary descriptive analysis identified the four most reported TRT with the highest incidence (*n*, %) over the intervention period. The ToxT approach was applied to these variables to analyse the data at discrete timepoints (weekly) between groups over time. The SAS code developed at the MAYO clinic generates plots using repeated measures modelling and area-under-curve (AUC) analysis [22]. Between-group or longitudinal differences within groups for continuous variables were analysed using *t*-tests and Kruskal–Wallis. Categorical variables were summarized as proportions and compared using chi-square tests or Fischer’s exact tests. Data were analysed using SAS and SPSS software (version 20.0, IBM, Armonk, NY, USA), while statistical significance was determined at *p* < 0.05.

## Results

A total of 43 participants were recruited and completed baseline assessments. Four participants withdrew from the study prior to the 10-week assessment due to non-compliance to the study protocol and/or disease progression. Anthropometric, diagnostic and treatment characteristics are presented in Table 1. No significant difference (*p* ˃ 0.05) was observed between groups for age, height, weight, body mass index (BMI), diagnosis or treatment characteristics at baseline.

**Table 1:** Characteristics of participants in the exercise (EG) and control (CG) groups, including prescribed treatments

Characteristic	EG (*n* = 21)	CG (*n* = 22)	*p* value
Patient demographics			
Age (years), mean (SD)	21.9 (3.0)	20.3 (2.7)	0.07
Males, *n* (%)	15 (68%)	12 (57%)	0.46
Cancer diagnosis			
Hodgkin lymphoma (*n*)	6	5	0.27
Sarcoma (*n*)	2	8	
CNS tumour (*n*)	6	5	
Germ cell tumour (*n*)	4	1	
Leukaemia (*n*)	2	2	
Melanoma (*n*)	1	0	
Burkitt lymphoma (*n*)	1	0	
Treatment protocol			
Low/moderate intensity			0.41
ABVD (*n*)	3	2	
Temozolomide (*n*)	4	5	
PCV (*n*)	1	1	
BEP (*n*)	4	1	
Ipilumamap/nivolumab (*n*)	1	0	
High intensity			
MAP (*n*)	0	2	
MAID (*n*)	1	0	
VDC/IE (*n*)	1	3	
ARST1431 (*n*)	0	3	
Escalated BEACOPP (*n*)	3	3	
CODOX M/IVAC (*n*)	1	0	
AML induction (7–3 Ida) + consolidation (5–2 Ida) (*n*)	2	1	
ALL-09 (*n*)	0	1	
Treatment intensity			
Low/moderate intensity	13	9	0.29
High intensity	8	13	
Radiation treatment			
Radiation (*n*)	6	12	0.08
Dose of radiation (Gy), mean (SD)	17.05 (27.70)	31.26 (27.79)	0.10
Body surface area (m^2^), mean (SD)	1.88 (0.22)	1.91 (0.18)	0.49

### Patient-reported toxicities

All participants reported a number of TRTs over the intervention period. When categorised to reflect the maximal worst grade experienced at any point throughout the intervention (Grade 0, Grade 1–2 and ≥ Grade 3) the CG reported significantly more severe fatigue (≥ Grade 3) than EG. No other differences between groups were evident (*p* > 0.05) (Table 2).

**Table 2:** Comparison of maximal worst grade toxicities reported for patient-reported toxicities between groups

	Exercise group (*n* = 21)		Control group (*n* = 22)		*p* value
	*n*	%	*n*	%	
Fatigue					
Grade 0	0	0	0	0	0.050
Grade 1–2	18	85.7	13	59.1	
Grade 3–4	3	14.3	9	40.9	
Nausea					
Grade 0	2	9.5	3	13.6	0.157
Grade 1–2	19	90.5	17	77.3	
Grade 3–4	0	0	2	9.1	
Pain					
Grade 0	2	9.5	2	9.1	0.674
Grade 1–2	16	76.2	17	77.3	
Grade 3–4	3	14.3	3	13.6	
Mood disturbances					
Grade 0	2	9.5	1	4.5	0.916
Grade 1–2	17	80.6	19	86.4	
Grade 3–4	2	9.5	2	9.1	
Diarrhoea					
Grade 0	13	61.9	12	54.5	0.760
Grade 1–2	8	38.1	10	45.5	
Grade 3–4	0	0	0	0	
Constipation					
Grade 0	9	42.6	8	36.4	0.760
Grade 1–2	12	57.1	14	63.6	
Grade 3–4	0	0	0	0	
Vomiting					
Grade 0	9	42.9	15	68.2	0.129
Grade 1–2	12	57.1	7	31.8	
Grade 3–4	0	0	0	0	
Peripheral neuropathy					
Grade 0	6	28.6	8	36.4	0.632
Grade 1–2	15	71.4	13	59.1	
Grade 3–4	0	0	1	4.5	
Dyspnoea					
Grade 0	4	19.0	3	13.6	0.651
Grade 1–2	15	71.4	18	81.8	
Grade 3–4	2	9.5	1	4.5	
Mucositis					
Grade 0	9	42.6	11	50.0	0.650
Grade 1–2	11	52.4	10	45.5	
Grade 3–4	1	4.8	1	4.5	
Insomnia					
Grade 0	5	23.8	4	18.2	0.721
Grade 1–2	13	61.9	16	72.7	
Grade 3–4	3	14.3	2	9.1	

### Toxicity over time (ToxT)

**Fig. 2:**
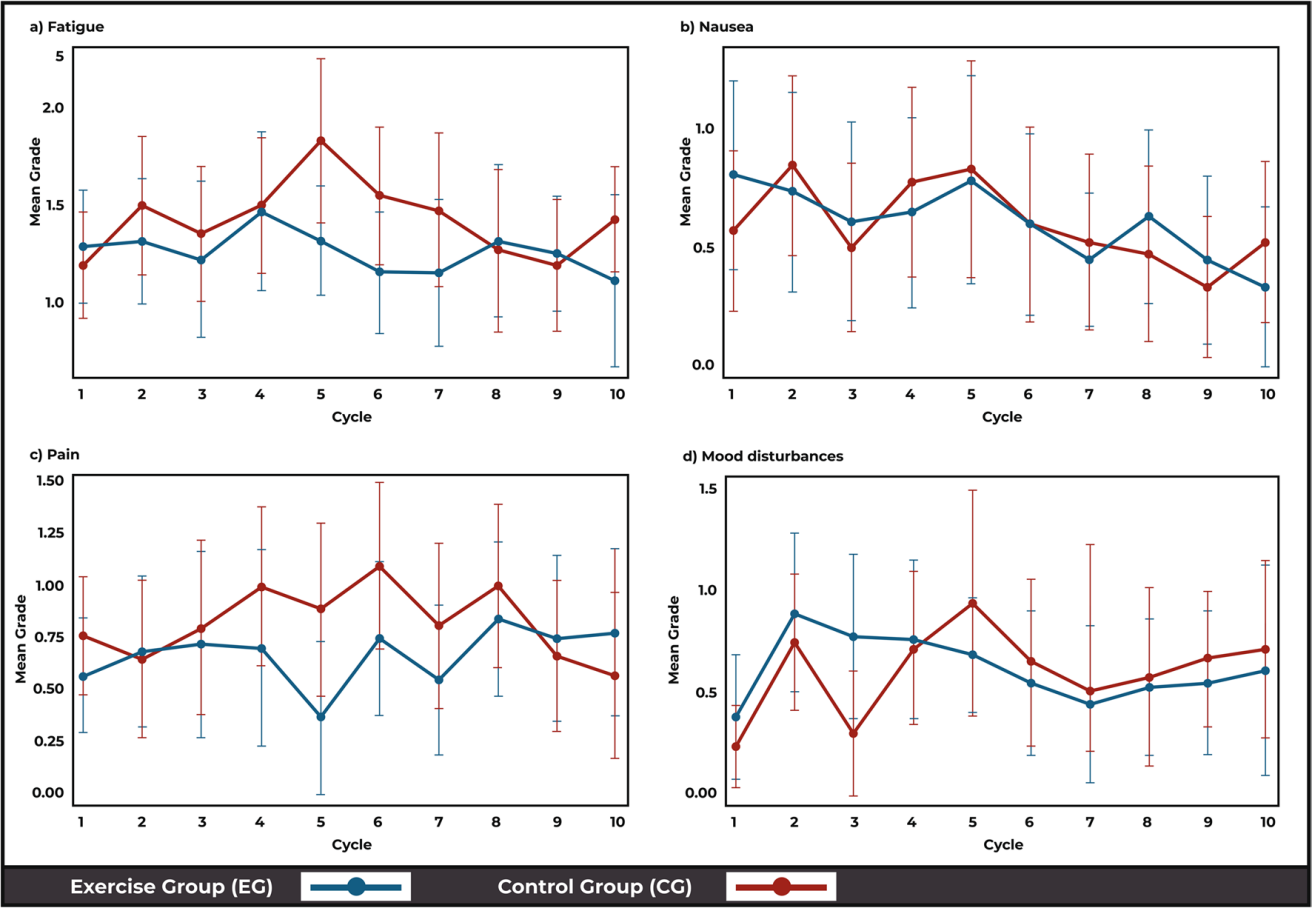
Stream plot of mean fatigue (**a**), nausea (**b**), pain (**c**) and mood disturbance (**d**) toxicities over time in the exercise (EG) and control (CG) groups (± 95% CI)

**Fig. 3:**
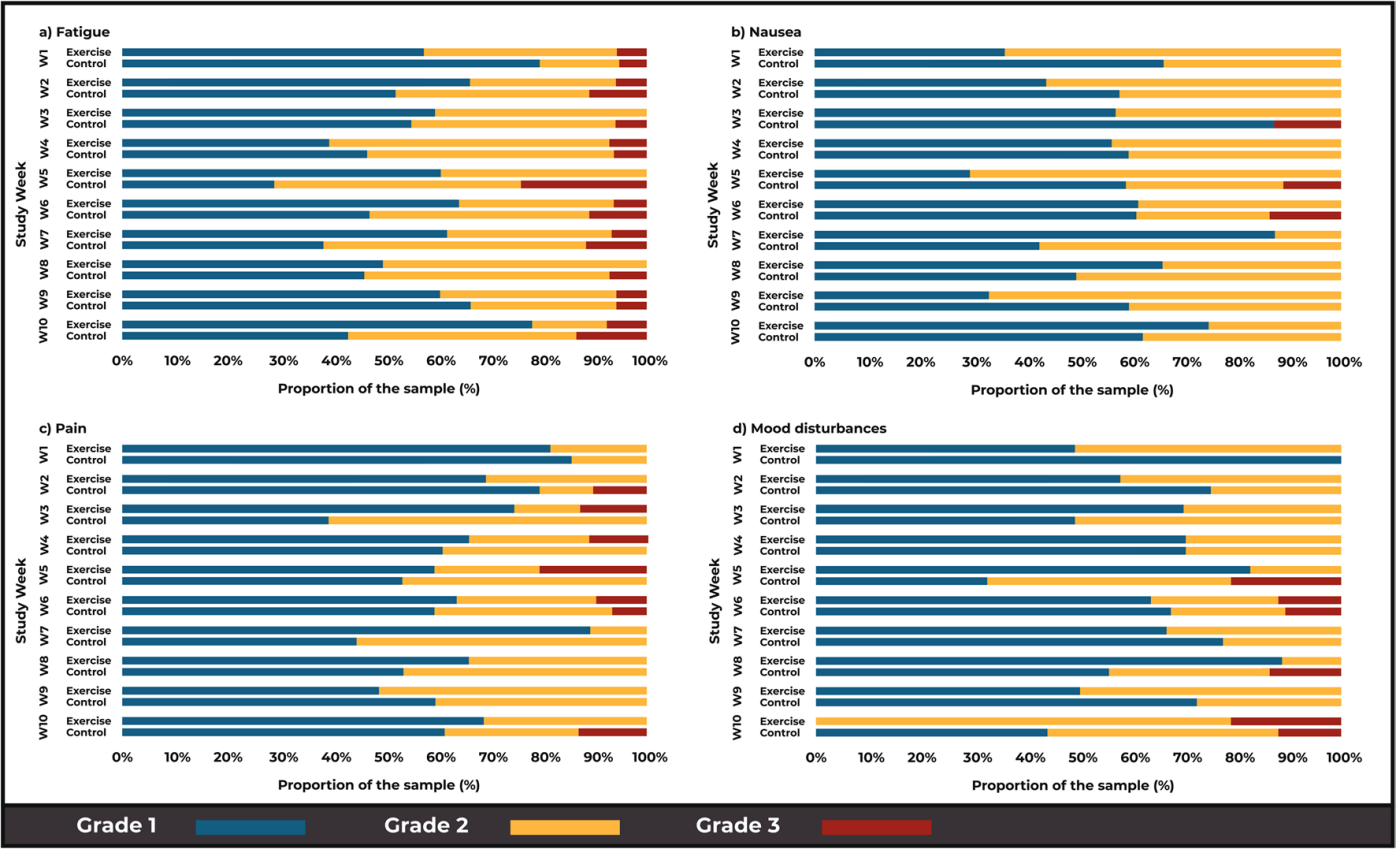
Stacked bar charts representing the frequency of recorded fatigue (**a**), nausea (**b**), pain (**c**) and mood disturbance (**d**) toxicities in the exercise (EG) and control (CG) groups as per CTCAE grading

The four commonest patient-reported TRTs in this both groups were fatigue, nausea, pain and mood disturbances. Using the ToxT approach there were no between-group differences in the mean grade of each of the four toxicities; fatigue (*p* = 0.25), nausea (*p* = 0.88), mood (*p* = 0.39) and pain (*p* = 0.92) over time (Fig. 2). Stream plots depict the mean grades over time and stacked bar charts reveal the frequency and proportion of each grade (1 to 4) of four common toxicities for both groups, over the intervention period. The stream plot for fatigue (Fig. 2a) demonstrates an increase in fatigue over time in the CG (24.6%, mean fatigue 1.14 in week 1 to 1.42 in week 10) and a decrease in the EG (29.3%, 1.33 in week 1 to 0.94 in week 10). Similarly, over the 10-week period, the CG demonstrated an increased incidence of severe fatigue, with participants in the CG having reported Grade 3 fatigue for all 10 weeks (100%), in comparison to the EG participants who experienced Grade 3 fatigue for only 70% of the intervention period (7 of 10 weeks) (*p* = 0.05) (Fig. 3a).

With respect to nausea, the mean toxicity grade decreased for both the EG (0.86 in week 1 to 0.17 in week 1) and CG (0.54 in week 1 to 0.45 in week 10) (Fig. 2b) stacked bar charts revealed that most of the nausea was Grade 1 or 2 in both groups over the 10-week intervention. However, the CG had reported Grade 3 nausea in weeks 3, 5 and 6, which was not evident in the EG at any point throughout the intervention (Fig. 3b).

Comparatively, for recorded pain, the EG had reported Grade 3 toxicities for five of the weeks, comparative to 2 weeks in the CG (Fig. 2c). However, stream plots revealed the mean pain toxicity over time appeared to be lower in the EG compared to the CG, although a slight decrease from baseline in the CG (0.75 in week 1 to 0.60 in week 10) and a slight increase in EG (0.61 in week 1 to 0.67 in week 10) was observed (Fig. 3c).

Finally, the mean mood disturbance toxicity appeared to increase over time in the CG (0.25 in week 1 to 0.70 in week 10) and decrease in the EG (0.50 in week 1 to 0.44 in week 10) (Fig. 2d). Furthermore, as with trends in fatigue and nausea, the CG had more occurrences of Grade 3 toxicity over time compared with the EG (EG 2/10 weeks, CG 4/10 weeks) (Fig. 3d). These results are descriptive in nature, and no statistically significant differences between groups were evident (*p* > 0.05).

**Fig. 4:**
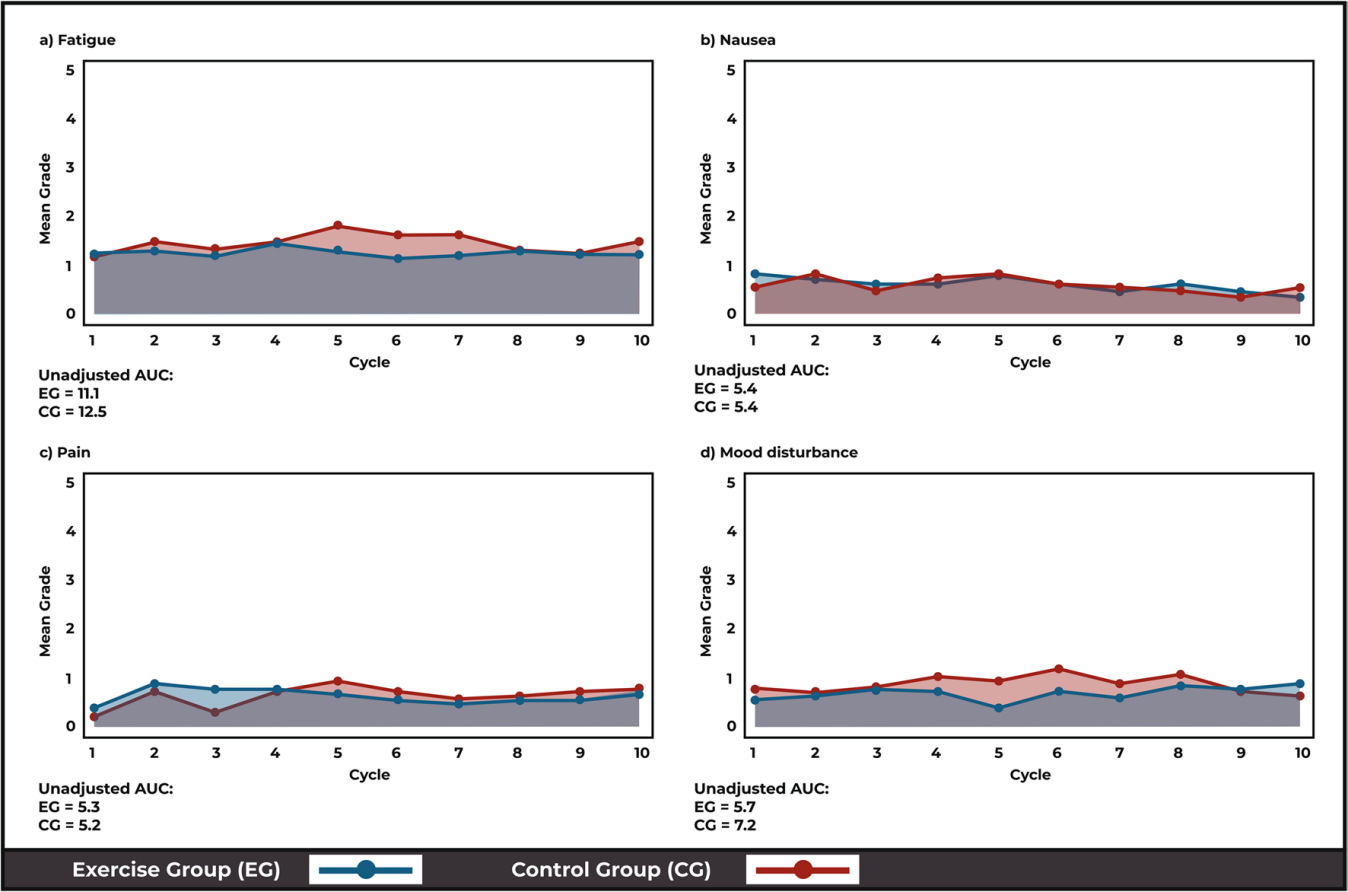
AUC analysis comparing recorded fatigue (**a**), nausea (**b**), pain (**c**) and mood disturbance (**d**) toxicities over time in the exercise (EG) and control (CG) groups

With respect to the AUC analysis, these results reflected a similar chronic burden of low-grade fatigue, nausea, mood disturbances and pain when calculated at the group level over time (Fig. 4). While no significant differences between groups were evident, the CG reported greater AUC for fatigue (CG:12.5; EG:11.1) and mood disturbances (CG:7.2; EG:5.7) over the intervention (*p* = 0.11 and *p* = 0.28, respectively) (Fig. 4a, d).

### Adverse events

Three participants (2:EG; 1:CG) were required to attend emergency departments for review following full blood count results emanating from this study. Two participants (1:EG; 1:CG) required platelet transfusions and one (EG) required a whole blood transfusion, each of which may have otherwise been missed without patients adhering to this study protocol. An additional participant was required to attend the emergency department and was subsequently admitted to the hospital for an immunotherapy-related headache. Mild delayed onset muscle soreness (DOMS) was reported in four participants following baseline assessment. No other adverse events were recorded.

## Discussion

There is now strong evidence supporting the benefits of exercise to mitigate numerous TRTs in a range of adult cancer cohorts [12, 13]. Despite this, evidence in AYAs remains limited, with respect to both the impact of exercise on TRTs as well as the collection and reporting of TRTs in this cohort. To our knowledge, the current study is the first RCT to investigate the impact of an exercise intervention on patient-reported TRTs as collected using conventional CTCAE reporting methods. Additionally, this study is the first to report the trajectory of toxicities throughout treatment in AYA cancer patients. The most important finding of the current study was that exercise reduces the severity of fatigue in AYAs undergoing treatment. Additionally, the longitudinal analysis has provided more nuanced insight into the symptom burden experienced by AYAs undergoing cancer treatment.

Initially, when adopting conventional the maximal worst grade approach, results revealed the CG experienced a significantly greater incidence of severe fatigue (≥ Grade 3) compared with the EG. This fatigue, categorised as being not relieved by rest and limiting self-care activities, likely interrupted their daily functioning and has the potential to impact their ability to meet developmental milestones [23]. Supporting previous evidence, this study demonstrated the benefits of exercise in reducing fatigue in AYAs undergoing treatment [23]. No other significant differences were identified between groups for any toxicities using this conventional analysis approach.

Regardless of age, diagnosis or specific treatment protocol, patients undergoing cancer treatment often experience a myriad of TRTs [24]. Standard toxicity reporting in clinical trial publications reports the maximal worst grade experienced at a single timepoint throughout treatment with a consensus that ≥ Grade 3 toxicities are of the greatest clinical relevance due to their potential life-threatening nature [25]. However, in recent years it has been recognised that the arbitrary cutoff of ≥ Grade 3 fails to recognise the development of toxicities over time as well as any persistent or chronic lower grade toxicities which often have deleterious impacts on patients’ abilities to tolerate treatment and their overall QOL [10]. Novel statistical analysis may better detect these lower grade but clearly troubling side effects [25]. Thanarajasingam et al. [21] developed an innovative approach to measuring toxicities that better reflects the “area under the curve” for low grade, but chronic, toxicities that may be clinically significant [21]. Therefore, through the adoption of longitudinal analysis using stacked bar charts and stream plots, unique insights into the possible interactions of exercise and TRTs were revealed.

Supporting previous research, the most common toxicities reported in the current cohort were fatigue, pain, nausea and mood disturbances [5, 6]. The ToxT approach was applied to these variables to provide insight into the patient experience of these toxicities over time [10]. Supporting the maximal worst grade results, the longitudinal analysis of fatigue revealed trends toward an increase in the incidence of severe fatigue throughout the weekly monitoring in the CG. Furthermore, there was a relative increase in mean fatigue over time in the CG with a comparable decrease evident in EG throughout the intervention period. These trends may also be apparent in the AUC results with a higher magnitude of fatigue reported in the CG, thus potentially limiting their ability to perform activities of daily living (ADLs). Collectively, this potential amelioration of fatigue as a result of exercise may contribute to AYAs being able to maintain their normal ADLs and remain engaged in treatment and psychosocial pursuits.

Chemotherapy-induced nausea has been reported to affect 18–81% of AYAs undergoing treatment [26]. Previous research has reported insufficient evidence to support the efficacy of exercise in mitigating nausea and vomiting [27]. However, Shim et al. (2019) reported smaller proportions of exercise group participants experiencing higher grade (Grades 3 and 4) nausea when compared with controls [28]. This was similarly reflected in our study with the EG not reporting any occurrences of Grade 3 nausea during the intervention compared to the CG who reported Grade 3 nausea for 30% of the intervention period. Given that poorly managed nausea can lead to dehydration, malnutrition and anorexia, the ability for the EG to avoid severe nausea may prevent them from experiencing such deleterious impacts and subsequently poorer outcomes [29]. Collectively however, longitudinal analysis revealed trends toward a reduction in nausea in both groups over time suggesting improved clinical management of nausea with effective anti-emetic regimes with each cycle of treatment. This effective management may in part be attributed to the rigorous recording of TRTs as result of enrolment in this study which was relayed to treating teams.

Previous research has reported more than 30% of AYAs undergoing treatment experience depression and anxiety symptoms [30]. The current results reflected this, with mood disturbances including anxiety and depression being the most cited TRTs. High-quality evidence now supports the use of exercise to reduce depression and anxiety in cancer cohorts [13]. In our longitudinal analysis, the EG appeared to experience less severe mood disturbances over the course of the intervention. AUC results potentially support this, with a lower magnitude of mood disturbances in the EG over time. Furthermore, the mean mood disturbances experienced by the EG appeared to reduce over time, with a comparative increase in the CG. This suggests that supervised exercise has the potential to help stabilise mood in AYAs undergoing cancer treatment.

Compared to the previous TRTs mentioned, our EG demonstrated trends toward more severe pain toxicity during the intervention period compared with the CG. This contrasts with a recent meta-analysis which reported significant favourable effects of exercise in mitigating pain, compared to a pooled control sample [31]. Given that pain toxicity in our study was a broad item, not capturing the specific cause of the pain, these results are difficult to interpret. We speculate that these results may be reflective of the delayed onset muscle soreness (DOMS) experienced in the EG during the intervention, which was avoided in the CG due to lack of additional structured activity. More rigorous pain symptomatology monitoring is warranted to gain further insight into the cause of pain and potential impact exercise may have.

### Limitations

We acknowledge a number of limitations in our study. Firstly, results may be affected by the inability to blind study participants and a further inability to prevent the CG from exercising during the intervention. Additionally, as this was a heterogenous cancer-diagnostic and treatment sample, the scheduling and toxicity profile variations of different treatment protocols are difficult to account for and this may have confounded results. Non-compliance to the study protocol may have also confounded results with four participants withdrawing prior to the 10-week assessment, subsequently reducing strength of the sample. Furthermore, the COVID-19 pandemic impacted study recruitment, exercise adherence and toxicity monitoring. Finally, this study was a secondary outcome of a larger RCT on which the sample size was powered to detect changes in Vo2peak (primary outcome). Given the large number of variables contributing to the toxicity monitoring, the current study was likely underpowered to detect significance in such a large number of outcome variables. Based on the trends observed in the current study, future research should be conducted and powered appropriately to detect changes in toxicities specifically.

## Conclusion

This study demonstrated that a 10-week exercise intervention reduces the severity of fatigue in AYAs undergoing cancer treatment. Longitudinal analysis also revealed positive trends supporting the benefits of exercise in potentially decreasing the severity of mood disturbances for AYAs. Additionally, it provided insights into the symptom burden experienced by AYAs which would have otherwise been missed using traditional analysis methods and further supports the notion that rigorous monitoring and reporting of TRTs is needed for this cohort. Future research should apply these methods to improve understanding into persistent lower grade toxicities often negatively impacting patient QOL as the primary outcome. While these trends must be interpreted with caution due to their lack of statistical significance, they provide insight into the possible reduction of symptom burden and should be investigated in a larger homogenous sample.

### Supplementary Information

Below is the link to the electronic supplementary material.

Supplementary file1 (DOCX 13 KB)

## Data Availability

The data that support the findings of this study are not openly available due to privacy reasons and are available from the corresponding author upon reasonable request.
