# Supplementary material for: The potential impact of exercise upon symptom burden in adolescents and young adults undergoing cancer treatment
Source: Support Care Cancer. 2024 Apr 17;32(5):293. doi: 10.1007/s00520-024-08497-0 (PMC11023984; doi:10.1007/s00520-024-08497-0)
Supplement: Supplementary file 1 — Supplementary file1 (DOCX 13 KB) [file 520_2024_8497_MOESM1_ESM.docx]

Supplementary material 1: Stratification strategy of most common adolescent and young adult (AYA) cancer diagnoses based on treatment regimen.

| **Low/Moderate intensity treatment diagnoses** | **High intensity treatment diagnoses** |
| --- | --- |
| Hodgkin lymphoma  Germ cell tumours  Gynaecological tumours  Non-Hodgkin lymphoma (excluding Burkitt)  Melanoma | Acute lymphoblastic leukaemia  Lymphoblastic lymphoma  Burkitt lymphoma  Soft tissue and bone sarcoma  Squamous cell carcinoma of the head and neck |
